# Guanylate-binding protein 5 is a marker of interferon-γ-induced classically activated macrophages

**DOI:** 10.1038/cti.2016.59

**Published:** 2016-11-02

**Authors:** Yukio Fujiwara, Yoshiyuki Hizukuri, Kyoko Yamashiro, Naoyuki Makita, Koji Ohnishi, Motohiro Takeya, Yoshihiro Komohara, Yasuhiro Hayashi

**Affiliations:** 1 Department of Cell Pathology, Graduate School of Medical Sciences, Faculty of Life Sciences, Kumamoto University, Kumamoto, Japan; 2 Asubio Pharma Co. Ltd, Kobe, Japan

## Abstract

Macrophage activation is the main immunological process occurring during the development of several diseases, and the heterogeneity of macrophage activation or differentiation has been suggested to be involved in disease progression. In the present study, we attempted to identify molecules specifically expressed on human classically activated macrophages (M1) to investigate the significance of the M1-like phenotype in human diseases. Human monocyte-derived macrophages were differentiated into M1, M2a, M2b and M2c phenotypes, and also M1(−) (the M1 phenotype differentiated with interferon-γ) to eliminate the strong effects of lipopolysaccharides (LPS) on the gene expression profile. The gene expression profiles of those macrophage phenotypes were analyzed by a cDNA microarray analysis and were used for a bioinformatics examination to identify the markers of the M1 phenotype that are expressed in both M1 and M1(−). The gene expression profiles of murine macrophages were also evaluated. We identified guanylate-binding protein 5 (GBP5), which is associated nucleotide-binding domain and leucine-rich repeat containing gene family, pyrin domain containing 3 (NLRP3)-mediated inflammasome assembly in the M1 macrophages of both humans and mice. Notably, the expression of GBP5 protein was detected in cultured M1(−) as well as in M1 macrophages by western blotting, which means that GBP5 is a more generalized marker of the M1 phenotype compared with the M1 markers that can be induced by LPS stimulation. GBP5 is a useful candidate marker of the M1 phenotype.

Macrophages are detected in almost all organs, and macrophage activation is the main immunological process occurring during the development of several diseases. The heterogeneity of macrophage activation or differentiation was suggested in the late 1990s on the basis of differences in surface markers or nitric oxide/ornithine production, and activated macrophages have been suggested to be broadly divided into classically activated macrophages (M1) and alternatively activated macrophages (M2). M1 cells preferentially produce proinflammatory molecules including nitric oxide, interleukin-12 (IL-12), CXCL9, CXCL10, CXCL11 and reactive oxygen species, whereas M2 cells express anti-inflammatory molecules including ornithine, IL-10, CCL17, CCL18, CCL22 and scavenger receptors.^1, 2, 3, 4, 5^

Recently, studies using animal disease models have indicated that M1-like cells tend to be involved in metabolic syndromes including atherosclerosis and insulin resistance via the secretion of inflammatory molecules. In contrast, M2-like cells tend to be associated with tissue remodeling, immunosuppression, angiogenesis and tumor progression. In human diseases, the pathophysiological involvements of M2 cells have been under investigation because CD163, CD204 and CD206 are widely used as reliable markers for M2 polarization. In human malignant tumors, an increased number of CD163- or CD204-positive tumor-associated macrophages has been demonstrated to be associated with high-grade histological malignancy and a worse clinical prognosis.^6^ In human lung diseases, the increased expression of M2-related molecules in alveolar macrophages is linked to the advance of diseases such as idiopathic pulmonary fibrosis, chronic obstructive pulmonary disease and allergic asthma.^7, 8, 9^ M2-related molecules are additionally upregulated in adipose tissue macrophages in obese individuals and are associated with insulin resistance.^10^

However, few studies have investigated the role of the M1 phenotype in human diseases because of the lack of suitable antibodies available for use in immunohistochemical analysis. Therefore, in the current study, we attempted to identify the molecules that are specifically changed in M1-like macrophages.

## Results

### Expression patterns of general M1 marker genes in various subtypes of human macrophages

**Figure 1:**
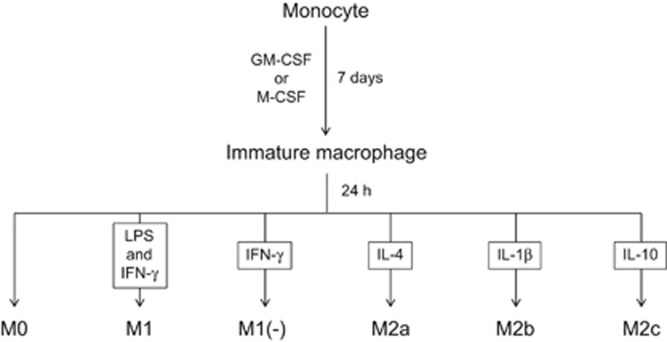
The inducing methods of each macrophage subtype.

**Figure 2:**
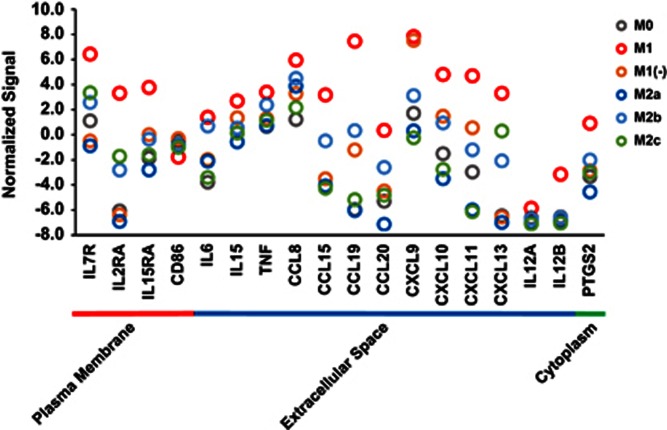
The expression signals of general M1 marker genes in various subtypes of human macrophages. Normalized signals (log base 2 and the 75th percentile signal value as 0) of general M1 marker genes are shown as gray (M0), red (M1), orange (M1(−)), blue (M2a), sky blue (M2b) and green (M2c) circles (mean, *n*=2). Those genes were categorized according to localization of their proteins retrieved from IPA.

Human macrophages were differentiated into the M1, M1(−), M2a, M2b and M2c subtypes as described in the Methods section and Figure 1, and DNA microarray analysis was performed to investigate the genes specifically expressed in M1 macrophages. The expression signals of M1 marker genes summarized in a previous review^11^ were extracted to confirm their high expression in our experiment (Figure 2). Except for CD86, the expression of these M1 marker genes were the highest in the M1 subtype. The strong expression of tumor necrosis factor-α, IL-12 and IL-6 in M1 macrophages was also confirmed at the protein level using a BioPlex Multiplex System (Miltenyi Biotec, Bergisch Gladbach, Germany) (Supplementary Figure 1). These data indicated that a typical M1 subtype was generated in our experiment.

**Figure 3:**
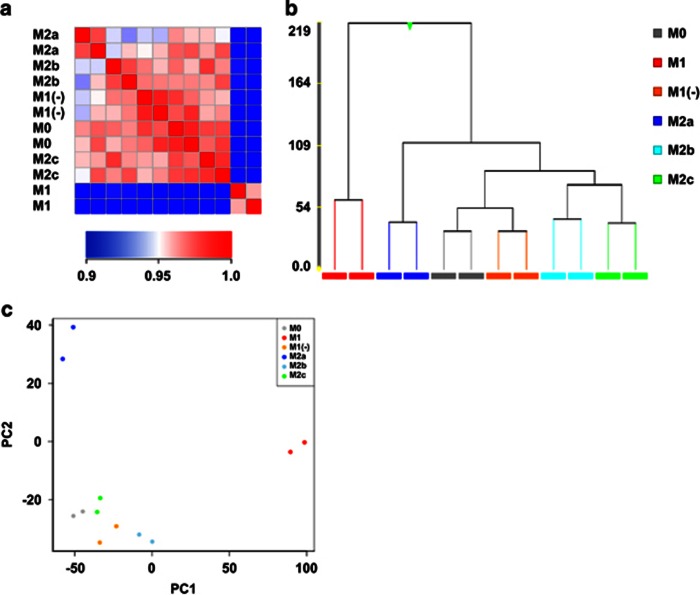
The expression profiles in human macrophage subtypes. Pearson's correlation coefficient between gene expression data of macrophage subtypes (*n*=2). The color of each cell represents the scores of subtype–subtype expression correlations (**a**). The hierarchical clustering of the gene expression data of macrophage subtypes. The similarity measure used was Euclidean and the linkage rule used was Ward's methods (**b**). The principal component analysis of the gene expression data of the macrophage subtypes. The x and y axes indicate the first and second principal components, respectively (**c**).

The data from the DNA microarray were analyzed to comprehend the general outline of expression profiles of macrophage subtypes. The correlation coefficient matrix of each subtype (Figure 3a) showed that M1 had a distinct and different profile, whereas the M1(−), M2b and M2c subtypes had profiles similar to that of M0 (macrophage without any stimulation), and these correlation coefficients were >0.95. The hierarchical clustering (Figure 3b) additionally showed that M1 occurred in its own cluster. On the other hand, M1(−) showed a more similar profile to M0, M2b and M2c compared with M1 (Figure 3a) and occurred in the same cluster as M0 (Figure 3b). A principal component analysis showed that M1 and M2a were characterized by the first and second component, respectively, whereas M1(−), M2b and M2c had similar profiles to M0 (Figure 3c). A gene ontology (GO) analysis was also performed and showed that there was no noteworthy difference between M1 and M2 (Supplementary Table 1). These results suggested that lipopolysaccharides (LPS) had a strong influence in the gene expression of M1 and masked the important gene expression signature of M1(−), namely M1 differentiated with only interferon-γ (IFN-γ).

The specificity of the M1 marker genes were re-evaluated in consideration of the gene expression patterns of M1(−). Only CXCL9 and CXCL11 were more highly expressed in M1(−) as well as in M1 compared with that in M0, M2a, M2b and M2c, and many of the marker genes were not more highly expressed in M1(−) compared with that in M0, M2a, M2b and M2c (Figure 2). Because CXCL9 and CXCL11 are chemokines secreted in the extracellular matrix and are not suitable as marker genes in experiments using antibodies such as immunohistochemical analysis, marker genes suitable for human M1 and M1(−) were not identified among the general M1 marker genes.

### Extraction of M1 and M1(−) subtype-specific genes in human macrophages

**Figure 4:**
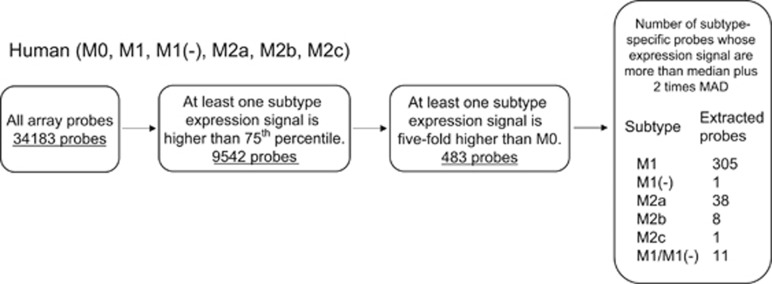
Extraction of M1 and M1(−) subtype-specific genes in human macrophages. The schema describes the workflow for extraction of subtype-specific upregulated genes in human macrophages. The number of probes upregulated in M1, M1(−), M2a, M2b and M2c is shown, as well as those in both M1 and M1(−) described as M1/M1(−).

The data obtained from DNA microarray experiments were further analyzed to identify candidate genes for M1 highly expressed both in M1 and M1(−). Criteria to extract candidate genes were set as follows: (1) at least one subtype expression signal is >75th percentile; (2) at least one subtype expression signal is fivefold higher compared with that of M0; and (3) subtype-specific gene expression is considered significant by criteria that expression signal is more than median plus two times median absolute deviation (MAD). Genes with a significant increase in expression in each subtype or both M1 and M1(−) were extracted as subtype-specific upregulated genes. The number of M1- and M1(−)-specific genes were 305 and 1, respectively, whereas the number of both M1 and M1(−) (hereafter described as M1/M1(−))-specific genes were 11 (Figure 4).

**Figure 5:**
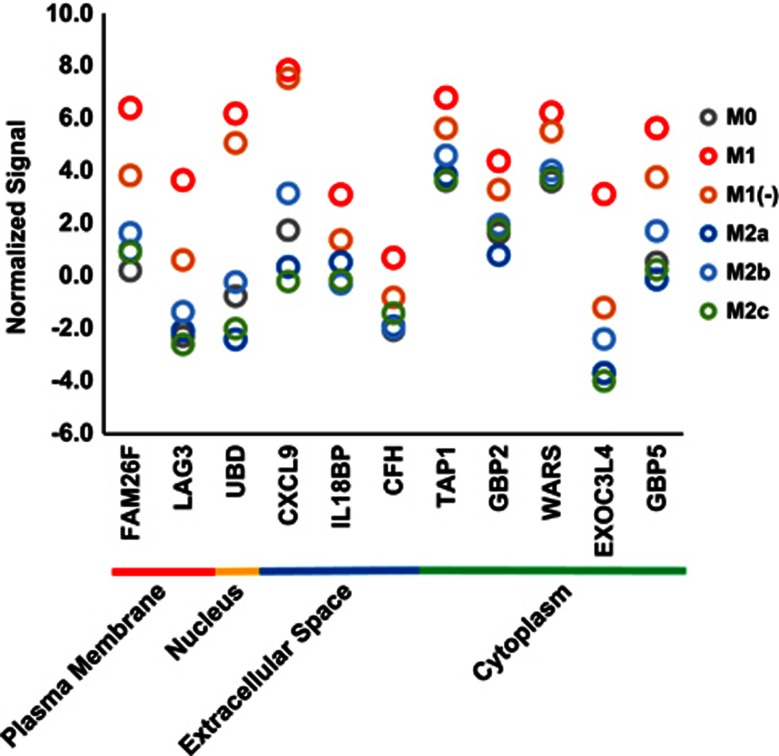
The expression signals of M1 and M1(−) subtype-specific genes in various subtypes of human macrophages. Normalized signals (log base 2 and the 75th percentile signal value as 0) of M1 and M1(−) subtype-specific genes are shown as gray (M0), red (M1), orange (M1(−)), blue (M2a), sky blue (M2b) and green (M2c) circles (mean, *n*=2). Genes were categorized according to the localization of their proteins retrieved from IPA.

The expression signals of M1/M1(−) subtype-specific genes in various subtypes of human macrophages are depicted in Figure 5. The gene expression of CXCL9 and the other 10 genes in M1(−) were almost equal to or less than those in M1. However, they were consistently higher compared with that in the subtypes other than M1 and M1(−). The expression signals of *FAM26F, LAG3, UBD, CXCL9* and *GBP5* (guanylate-binding protein 5) in M1 and M1(−) were at least four times higher (difference of normalized expression signals >2) compared with those of the other subtypes (Table 1). Thus, we were interested in *FAM26F, LAG3, UBD, CXCL9* and *GBP5* as M1/M1(−) subtype-specific genes.

### Selection of M1/M1(−) subtype-specific marker genes in human macrophages

Generally, marker molecules are more suitable if they are expressed in both humans and mice. As we thought that marker genes should be expressed both in humans and mice, DNA microarray experiments were conducted to obtain the gene expression profiles of mouse macrophage subtypes and were analyzed as in the case of human macrophages. The number of M1/M1(−)-specific genes were 334 (Supplementary Figure 2). Relevant information for the 11 genes shown in Figure 5 is summarized in Table 1. Eight of the 11 genes were additionally extracted as M1/M1(−)-specific genes in mouse macrophages. It is suggested that the expression of these eight genes were preserved among species, at least for humans and mice. Among the five M1/M1(−) subtype-specific genes, *FAM26F*, *UBD*, *CXCL9* and *GBP5* were additionally M1/M1(−)-specific in mouse macrophages, whereas *LAG3* was not. Because CXCL9 is a chemokine that is secreted in the extracellular matrix, we did not consider *CXCL9* to be suitable as a marker gene. Thus, *FAM26F*, *UBD* and *GBP5* were selected as candidates of M1/M1(−) subtype-specific marker genes.

### Confirmation of higher expression of GBP5 in human M1 and M1(−) subtypes

**Figure 6:**
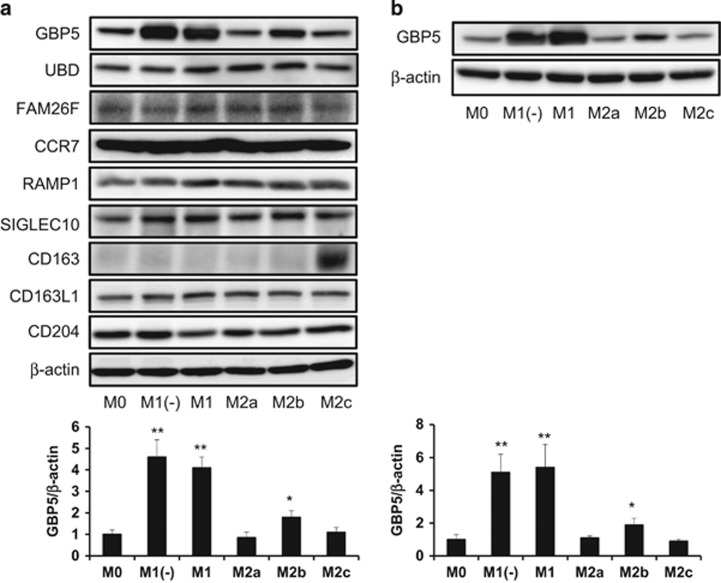
The protein expression of GBP5 in several human macrophage phenotypes. M-CSF- (**a**) or GM-CSF- (**b**) induced differentiated macrophages were incubated with several cytokines for 24 h, followed by the determination of the GBP5 or other protein and β-actin expression levels using a western blot analysis, as described in the Methods section. All experiments were repeated five times with almost identical results. The data are presented as the mean ± s.d. *P<0.05, **P<0.005 compared to M0.

**Figure 7:**
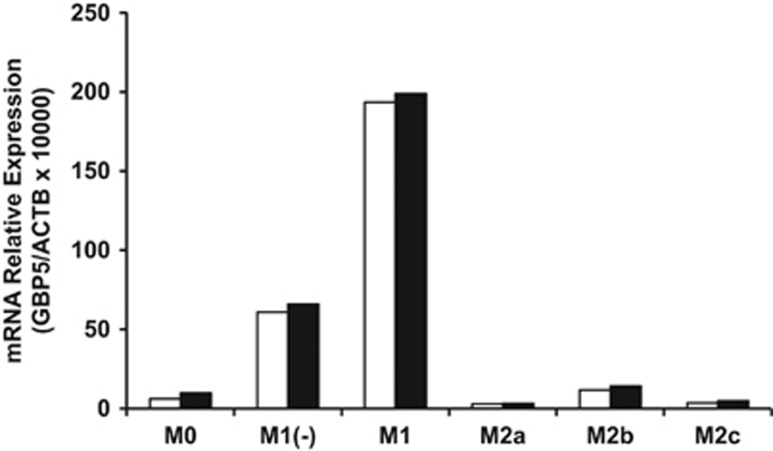
The gene expression of GBP5 in human macrophage subtypes. GBP5 gene expression in macrophage subtypes was determined using real-time PCR to confirm the results of the microarray. Higher GBP5 expression was confirmed in the RNA extracts of M1 and M1(−) macrophage subtypes used for DNA microarray experiments. Open and closed columns represents two different donors, and data of each column are mean of two replicates.

As *FAM26F*, *UBD* and *GBP5* were selected as candidates of M1/M1(−) subtype-specific marker genes, we next measured their protein expression in each macrophage phenotype by western blot analysis. As a result, the protein expression of *GBP5* in M1 and M1(−) was higher compared with that in the other subtypes, whereas that of *FAM26F* and *UBD* was not (Figure 6a). The protein expression of *GBP5* in M1 and M1(−) macrophages derived from granulocyte-macrophage colony-stimulating factor (GM-CSF)-induced human monocyte-derived macrophages (HMDMs) was additionally higher compared with that of the other subtypes (Figure 6b). IFNγ induced the expression of GBP5 in the HMDMs in a dose-dependent manner (Supplementary Figure 3). Furthermore, in murine macrophages, the protein expression of *GBP5* in M1 and M1(−) was also higher compared with that of the other subtypes (Supplementary Figure 4). To confirm higher expression at the mRNA level, the RNA extracts of human macrophage subtypes derived from two different donors used for DNA microarray experiments were analyzed using real-time PCR. The expression of GBP5 in M1 and M1(−) was higher compared with that of the other subtypes (Figure 7), thereby replicating the results of the microarray data. The enhanced expression of GBP5 mRNA in M1 and M1(−) derived from M-CSF- and GM-CSF-induced HMDMs (two and one donors, respectively) was additionally confirmed (data not shown). Thus, GBP5 is suggested to be a useful candidate marker of the M1 phenotype.

### CD163 is a useful marker of human M2c subtype

We additionally attempted to evaluate the M2 marker genes identified by means of DNA microarray experiments. RAMP1 and SIGLEC10 were suggested as M2a and M2b markers, respectively. CD163 previously reported to be the M2c marker^12^ and its related molecule, CD163L1, were additionally evaluated by means of western blotting using human macrophage lysates. Only CD163 was found to be specifically expressed on an expected subtype, M2c (Figure 6a). CCR7 and CD204 are well-known molecules used for M1 and M2 markers, respectively,^13, 14^ although these molecules were not identified in the present analysis. Interestingly, CCR7 and CD204 were detected on all subtypes of macrophages, and there was no difference in expression levels between macrophage subtypes (Figure 6a). These data indicate that CD163 is a reliable and useful marker for M2c in human macrophages.

## Discussion

The stimulation of macrophages with LPS and/or IFN-γ elicits several events such as the production of cytokines, chemokines and other communication signals important for the coordination of inflammatory responses.^15, 16, 17^ Interferon-regulatory factors (IRFs), transcription factors binding to the conserved virus response elements within the promoters of type 1 IFN genes, including IRF-1, IRF-5 and IRF-8, additionally induce the M1 phenotype in macrophages.^18^ It is known that the inflammatory induction of LPS is stronger than that of IFNs. In fact, the expression of general M1 marker genes were higher in M1 (LPS+IFN-γ-induced subtype) compared with in M1(−) (IFN-γ-induced subtype) in the current study, suggesting that M1(−) represents a mild inflammatory subtype, whereas M1 represents a severe inflammatory subtype. Therefore, the extraction of M1 and M1(−) subtype-specific genes in both humans and mice is important for the identification of useful candidate M1 markers in several types of studies, such as the elucidation of the role of M1 in human diseases. In the present study, the data of DNA microarray experiments were analyzed to obtain candidate genes for M1, which were highly expressed in both M1 and M1(−) in the case of human macrophages. As a result, five M1/M1(−)-specific genes (*GBP5*, *LAG3*, *UBD*, *CXCL9* and *FAM26F*) were selected as candidate M1 markers by the criteria of an at least four times higher signal compared with those in the other subtypes. However, *LAG3* was not extracted as a candidate gene for M1, which was highly expressed in both M1 and M1(−) in the case of mouse macrophages (Table 1). CXCL9, which is secreted to the extracellular matrix, was not thought to be suitable for a marker gene. Among the remaining candidates, only *GBP5* was specific to M1 and M1(−) at the protein level (Figure 6).

*GBP5* belongs to the family of IFN-γ-induced p65 GTPases, which are well known for their high induction by proinflammatory cytokines, and has seven members in the human genome.^19^ This family of guanylate-binding proteins was originally identified by its ability to bind to immobilized guanine nucleotides with similar affinities for GTP, GDP and GMP.^20, 21^ GBP5 protein highly expressed in mononuclear cells.^22^ It is reported that GBP5 carries a C-terminal CaaX-prenylation signal, which increases the membrane affinity of proteins, and that its prenylation is required for membrane association.^23^

It is known that GBP5 is an important mediator of inflammatory immune response. Loss of GBP5 function in a knockout mouse model results in impaired host defense and inflammatory response as GBP5 facilitates nucleotide-binding domain and leucine-rich repeat containing gene family, pyrin domain containing 3 (NLRP3)-mediated inflammasome assembly.^24^ Mice lacking functional GBP5 had significantly reduced neutrophil recruitment in response to peritonitis. In addition, these knockout mice had increased bacterial burdens, severely reduced CD11b^+^ cells in mesenteric lymph nodes and noticeable weight loss in response to *Listeria monocytogenes* infection.^24^ Furthermore, GBP5 expression was upregulated in response to Epstein–Barr viral infection.^25^ In addition, GBP5 is sufficient to induce a heightened susceptibility of RAW 264.7 cells to Salmonella-induced pyroptosis, and the endogenous expression of GBP5 is important for this phenomenon,^26^ thus indicating that GBP5 has an important role in the host defense against *Salmonella enterica* serovar Typhimurium and perhaps other invasive bacterial pathogens.^26^ Those reports suggest that GBP5 is an important mediator of inflammatory macrophages.

Few studies have investigated the role of M1 phenotype in human diseases owing to the lack of both suitable antibodies and M1 phenotype markers, which are available for immunohistochemical analysis. Previous studies reported that CD68^+^RBP-J^+^ and CD68^+^HLADR^+^ are M1 phenotype markers.^27, 28, 29^ However, these markers are not clearly available as M1/M1(−) marker as they were not extracted as M1 and M1(−) subtype-specific genes in our present study. Although CCR7 has additionally been considered as an M1 marker^13^ and was specific to M1 at the mRNA level in our study (data not shown), CCR7 was not so specific to M1 at the protein level (Figure 6a). GBP5 selected as candidate M1/M1(−) markers in the present study might be useful as a M1 marker in humans and mice. However, we could not identify the commercial antibodies that specifically react to human GBP5 by means of immunohistochemical analysis (unpublished data). Therefore, the potential advantage of this marker is restricted to western blot analysis at this time, and available specific anti-GBP5 antibody in immunohistochemical analysis is needed in the future. On the other hand, CD163 is a reliable and useful marker for the human M2c phenotype in both western blotting (Figure 6a) and immunohistochemical analysis.^12^

Collectively, the significance of the present study is that we used transcriptome analysis and found GBP5 as a novel candidate of human M1 marker genes, which was specifically upregulated in both M1 and M1(−) from candidate genes and most of which were upregulated only in M1, suggesting that they were upregulated by LPS stimulation.

## Methods

### Reagents

Recombinant mouse cytokines (M-CSF, IFN-γ, IL-4, IL-1β and IL-10) were purchased from R&D Systems (Minneapolis, MN, USA). Recombinant human cytokines (GM-CSF, M-CSF, IFN-γ, IL-4, IL-1β and IL-10) were purchased from Wako (Osaka, Japan).

### Human macrophages

Peripheral blood mononuclear cells were acquired from healthy volunteer donors; written informed consent was obtained from all donors. This study was approved in Ethics Committee for Epidemiological and General Research at the Faculty of Life Science, Kumamoto University (Kumamoto, Japan; approval number: 486) and Ethical Evaluation Committee on Human Tissue and Other Human Material Research at the Asubio Pharma Co. Ltd (Kobe, Japan; approval number: E-11-018 and HT-12-024). CD14^+^ monocytes were purified from peripheral blood mononuclear cells via positive selection using magnetic-activated cell sorting technology (Miltenyi Biotec, Bergisch Gladbach, Germany) and were cultured with GM-CSF (10 ng ml^−1^) or M-CSF (50 ng ml^−1^) for 7 days to facilitate differentiation into macrophages. The differentiated macrophages were then used as HMDMs in the present study. Under these conditions, the cells contained >95% macrophages and were >98% viable (determined by trypan blue staining). To induce the macrophage subtypes (M1, M1(−), M2a, M2b and M2c), the macrophages were further stimulated for 24 h with LPS (10 ng ml^−1^)+IFN-γ (50 ng ml^−1^), IFN-γ (50 ng ml^−1^), IL-4 (10 ng ml^−1^), IL-1β (10 ng ml^−1^) and IL-10 (10 ng ml^−1^). The concentration of cytokines and LPS was based on a previous report^30^ with slight modifications. Control macrophages (M0) were prepared by incubating for 24 h without additional factors. The concentrations of TNF-α, IL-12 and IL-6 in the supernatants were measured using a BioPlex Multiplex System (Bio-Rad, Hercules, CA, USA). In the current study, the term ‘human macrophages' is used to denote M-CSF-induced HMDMs unless explicitly expressed as GM-CSF-induced HMDMs.

### Murine macrophages

C57BL/6J mice were purchased from Charles River Japan (Yokohama, Japan). All efforts were made to minimize suffering. This study was approved by the Ethics Committee for Animal Experiments of Asubio Pharma Co. Ltd (approval number: AEK-11-061). Bone marrow-derived macrophages were generated as described previously.^31^ Briefly, bone marrow cells from C57BL/6J mice were cultured in RPMI 1640 medium supplemented with 10% fetal bovine serum, 100 U ml^−1^ penicillin, 100 μg ml^−1^ streptomycin and M-CSF (50 ng ml^−1^) for 7 days to induce bone marrow-derived macrophages. To induce the macrophage subtypes (M1, M1(−), M2a, M2b and M2c), the macrophages were further stimulated for 24 h with LPS (10 ng ml^−1^)+IFN-γ (50 ng ml^−1^), IFN-γ (50 ng ml^−1^), IL-4 (10 ng ml^−1^), IL-1β (10 ng ml^−1^) and IL-10 (10 ng ml^−1^). The concentration of cytokines and LPS was based on a previous report.^31^ Control macrophages (M0) were prepared by incubating for 24 h without additional factors.

### Microarray and extraction of candidate genes

Macrophages derived from two different donors were used for microarray analysis. Total RNA was isolated using the RNeasy Mini Kit (Qiagen, Hilden, Germany). The transcriptional profile was evaluated using the Human Whole Genome ver. 2.0 arrays (G4845A; Agilent Technologies, Santa Clara, CA, USA) or Mouse Whole Genome ver. 2.0 arrays (G4846A; Agilent Technologies). Microarrays were scanned, and data extraction was conducted using Feature Extraction software version 9.5.1 (Agilent Technologies). The data were then analyzed using GeneSpring GX software version 12.6 (Agilent Technologies). Signal values were transformed to the log base 2 and the 75th percentile shift normalization performed, which used the 75th percentile signal value as 0. The probes with signals of <0 under all sample conditions were excluded; thus, the resultant probe list included a total of 10 479 probes. The analysis of Pearson's correlation coefficient was calculated and plotted to the heat map of correlations of subtypes. Hierarchical clustering was performed by Euclidean similarity measure and ward linkage. A principal component analysis was performed and the first and second principal components were plotted. We extracted the genes of each subtype that were upregulated to levels that were twofold higher compared with the levels in M0 and performed a GO analysis using DAVID.^32^ We extracted the level 3 GO terms associated with the biological process that had false discovery rate values <0.1. Microarray data have been deposited at GEO under accession number GSE85346.

### Extraction of subtype-specific upregulated genes

In each subtype (M0, M1, M1(−), M2a, M2b and M2c], the average signal of replicates were calculated. The probes whose signals were under the 75th percentile in all subtypes were filtered out of the data. Probes whose signals in all subtypes except M0 were ⩽5-fold of the signal in M0 were filtered out. Simple outlier detection method was performed by using MAD.^33^ MAD is a measure of dispersion or spread of quantitative data. For each probe, the log-transformed (base 2) signal values of all subtypes were used to calculate the median and MAD. Those probes with a signal larger than the median plus two times MAD were considered significant. Genes with a significant increase in expression in each subtype or both M1 and M1(−) were extracted as subtype-specific upregulated genes. The localization of M1- and M1(−)-specific genes was retrieved using Ingenuity Pathway Analysis (IPA; Qiagen, Redwood City, CA, USA, http://www.ingenuity.com/).

### Quantitative PCR

The RNA extracts of human macrophage subtypes that were used for the DNA microarray experiments were derived from two different donors and were analyzed by a real-time PCR (Figure 7). In additional experiments, the macrophage subtypes derived from M-CSF- and GM-CSF-induced HMDMs (two donors and one donor, respectively) were analyzed (data not shown). Briefly, cells were lysed, and total RNA was isolated using the RNeasy Mini Kit (Qiagen) according to the manufacturer's protocol. Total RNA was reverse transcribed using the SuperScript Vilo cDNA Synthesis Kit (Life Technologies, Carlsbad, CA, USA), and quantitative PCR was conducted using Taqman-based detection methods (universal probe library; Roche, Penzberg, Germany). β-Actin levels were used as the normalization control, and fold induction was calculated using the ΔCT methods. The primers used were GBP5 (NM_052942.3; forward, 5′-CAGGAACAACAGATGCAGGA-3′ reverse, 5′-TCATCGTTATTAACAGTCCTCTGG-3′ probe, no. 67), β-actin (NM_007393.3; forward, 5′-TCAACACCCCAGCCATGTA-3′ reverse, 5′-GTGGTACGACCAGAGGCATAC-3′ probe, no. 64).

### Western blot

M-CSF- or GM-CSF-induced differentiated macrophages that were derived from three donors were incubated with several cytokines. The degree of protein expression was determined by western blotting. Briefly, the solubilized cells were run on a 10% sodium dodecyl sulfate-polyacrylamide gel and transferred to a polyvinylidene fluoride transfer membrane (Millipore, Bedford, MA, USA). To detect GBP5 or each candidate gene product, the membranes were exposed to an anti-GBP5 antibody (8B12; Abcam, Cambridge, MA, USA), anti-CCR7 antibody (Y59; Abcam), anti-RAMP1 antibody (EPR10867; Abcam), anti-SIGLEC10 antibody (A01; Abnova, Taipei City, Taiwan), anti-FAM26F antibody (ab98871; Abcam), anti-UBD antibody (LS-C138099; LSBio, Seattle, WA, USA), anti-CD163 antibody (2G12; Abcam), anti-CD163L1 antibody (ab117250; Abcam) or anti-CD204 antibody (E5; TransGenic, Kobe, Japan), and visualized using a horseradish peroxidase-conjugated anti-mouse IgG antibody and anti-rabbit IgG antibody with an ECL western blotting detection reagent (GE Healthcare Bio-Sciences, Pittsburgh, PA, USA). The membranes were reblotted with an anti-β-actin antibody (C4) (sc-47778; Santa Cruz Biotech, Dallas, TX, USA) as an internal calibration control. The density of the bands was measured with the Imaging Gauge software program in an ImageQuant LAS 4000 system (GE Healthcare, Japan, Tokyo) and expressed as mean±s.d. (*n*=3).

### Statistics

All data are representative of two or three independent experiments. The data are expressed as the mean±s.d. Differences between the groups were examined for statistical significance using a non-repeated-measures analysis of variance. A *P*-value of <0.05 denoted the presence of a statistically significant difference.

## Figures and Tables

**Table 1:** List of M1 and M1(−) subtype-specific genes in human macrophages

*Symbol*	*Entrez gene name*	*Fold change*			*Extracted from mouse*
		*M1 vs M0*	*M1(*−*) vs M0*	*M1(*−*) vs the lower subtype next to M1(*−*)*	
*FAM26F*	*Family with sequence similarity 26, member F*	72.8	12.3	4.6 vs M2b	+
*LAG3*	*Lymphocyte-activation gene 3*	61.6	7.5	3.9 vs M2b	−
*UBD*	*Ubiquitin D*	123.4	56.8	39.2 vs M2b	+
*CXCL9*	*Chemokine (C–X–C motif) ligand 9*	68.4	55.4	20.9 vs M2b	+
*IL18BP*	*Interleukin-18-binding protein*	10.5	3.2	1.8 vs M2a	+
*CFH*	*Complement factor H*	6.7	2.4	1.5 vs M2c	−
*TAP1*	*Transporter 1, ATP-binding cassette, subfamily B (MDR/TAP)*	9	4	2.0 vs M2b	+
*GBP2*	*Guanylate-binding protein 2*	6.7	3.1	2.5 vs M2b	+
*WARS*	*Tryptophanyl-tRNA synthetase*	6.2	3.7	2.8 vs M2b	+
*EXOC3L4*	*Exocyst complex component 3-like 4*	113.2	5.7	2.3 vs M2b	−
*GBP5*	*Guanylate-binding protein 5*	34.6	9.5	4.1 vs M2b	+
