# Supplementary figures and images for: Guanylate-binding protein 5 is a marker of interferon-γ-induced classically activated macrophages
Source: Clin Transl Immunology. 2016 Nov 2;5(11):e111–. doi: 10.1038/cti.2016.59 (PMC5133363; doi:10.1038/cti.2016.59)

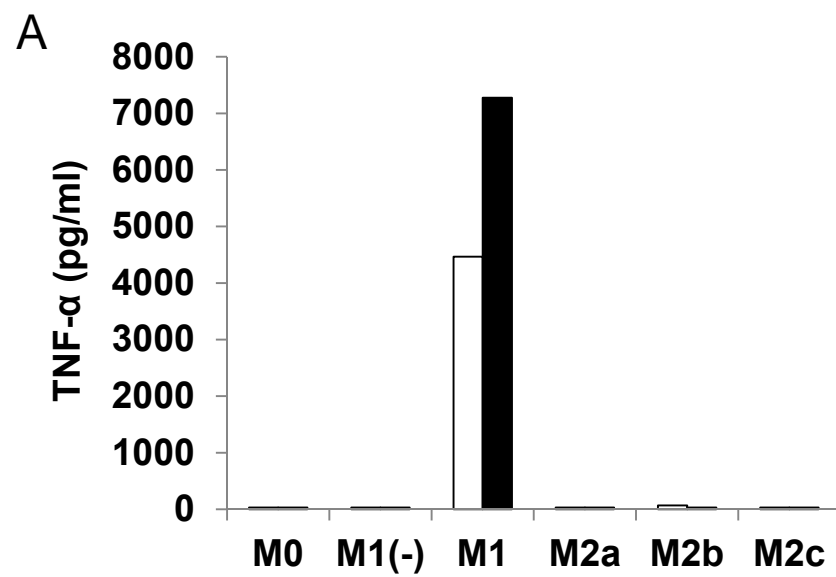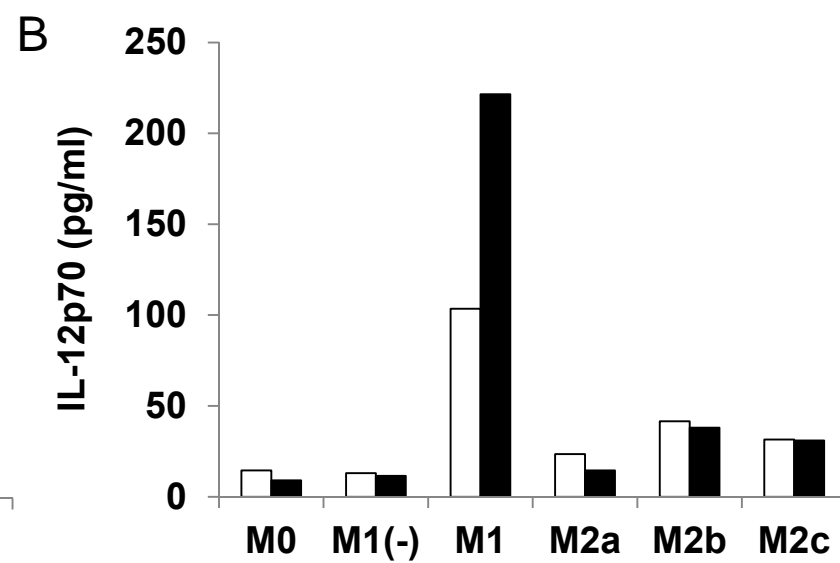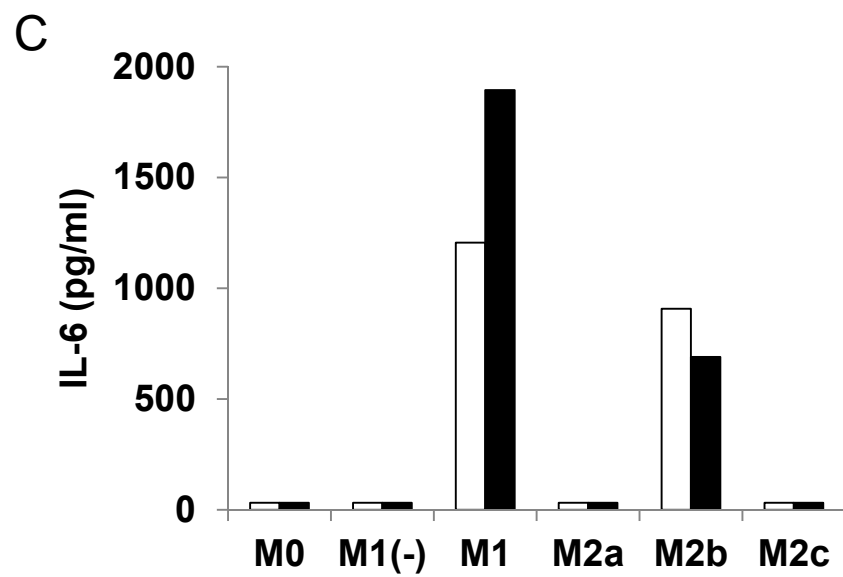

Supplement: Supplementary Figure 1 [file cti201659x1.pdf]

Mouse (M0, M1, M1(-), M2a, M2b, M2c)

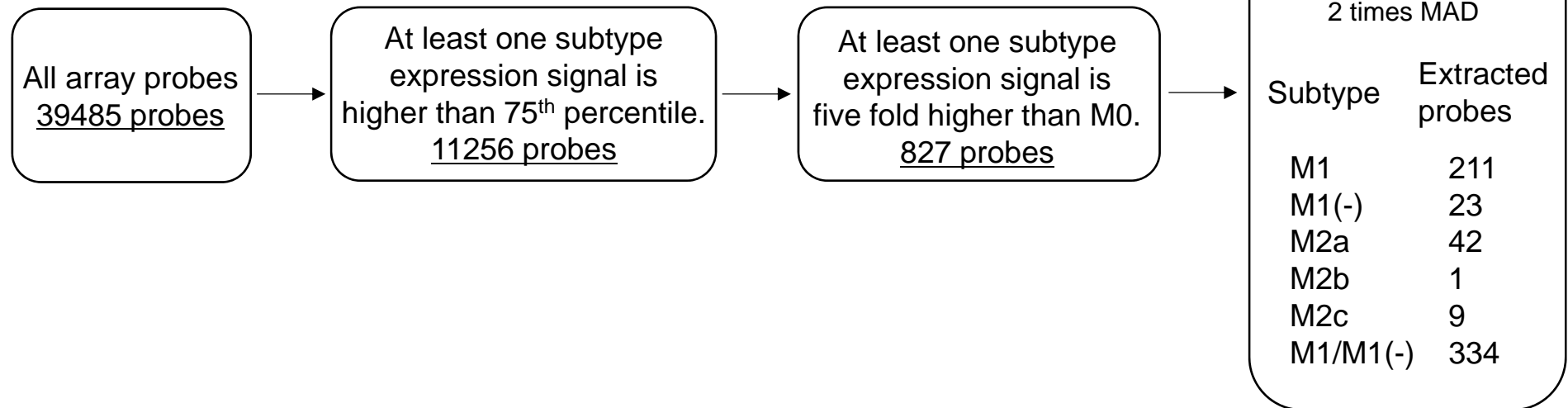

Supplement: Supplementary Figure 2 [file cti201659x2.pdf]

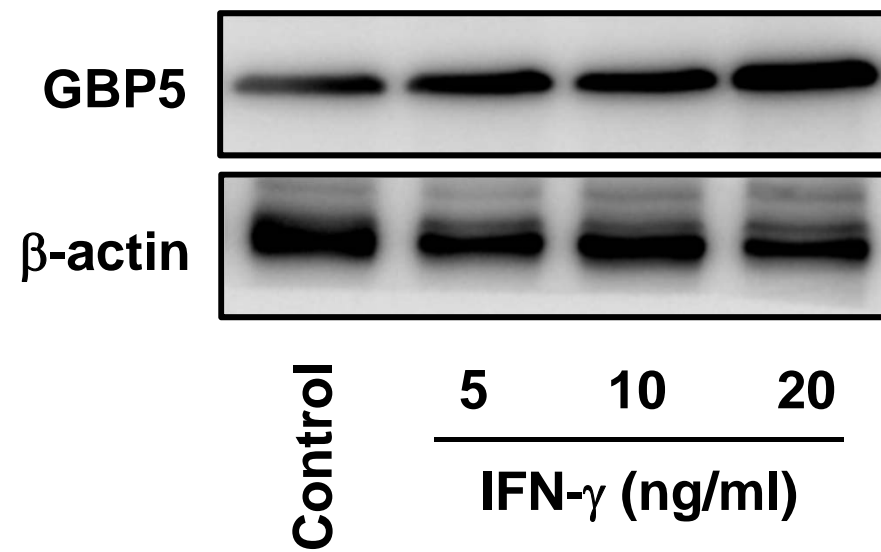

Supplement: Supplementary Figure 3 [file cti201659x3.pdf]

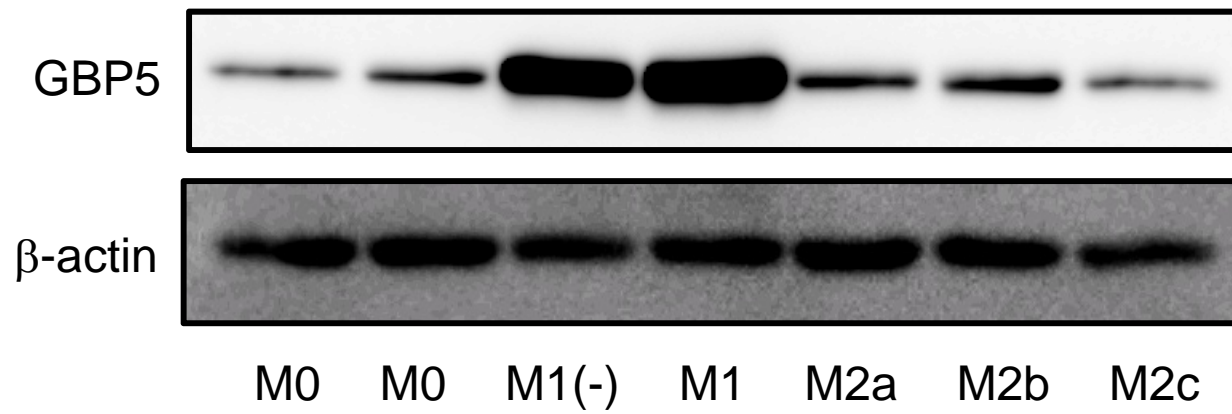

Supplement: Supplementary Figure 4 [file cti201659x4.pdf]
