# Supplementary material for: Guanylate-binding protein 5 is a marker of interferon-γ-induced classically activated macrophages
Source: Clin Transl Immunology. 2016 Nov 2;5(11):e111–. doi: 10.1038/cti.2016.59 (PMC5133363; doi:10.1038/cti.2016.59)
Supplement: Supplementary Table 1 [file cti201659x5.pdf]

M1
GO:0006952: defense response
GO:0009611: response to wounding
GO:0051707: response to other organism
GO:0010941: regulation of cell death
GO:0009615: response to virus
GO:0002682: regulation of immune system process
GO:0048583: regulation of response to stimulus
GO:0050776: regulation of immune response
GO:0012501: programmed cell death
GO:0048518: positive regulation of biological process
GO:0002684: positive regulation of immune system process
GO:0001817: regulation of cytokine production
GO:0010942: positive regulation of cell death
GO:0042330: taxis
GO:0006935: chemotaxis
GO:0048522: positive regulation of cellular process
GO:0048584: positive regulation of response to stimulus
GO:0031349: positive regulation of defense response
GO:0080134: regulation of response to stress
GO:0048519: negative regulation of biological process
GO:0050865: regulation of cell activation
GO:0050778: positive regulation of immune response
GO:0002694: regulation of leukocyte activation
GO:0010033: response to organic substance
GO:0042127: regulation of cell proliferation
GO:0002237: response to molecule of bacterial origin
GO:0060548: negative regulation of cell death
GO:0007626: locomotory behavior
GO:0050867: positive regulation of cell activation
GO:0043069: negative regulation of programmed cell death
GO:0045321: leukocyte activation
GO:0048523: negative regulation of cellular process
GO:0009617: response to bacterium
GO:0001819: positive regulation of cytokine production
GO:0002250: adaptive immune response
GO:0002696: positive regulation of leukocyte activation
GO:0002252: immune effector process
GO:0002218: activation of innate immune response
GO:0034097: response to cytokine stimulus
GO:0031497: chromatin assembly
GO:0002683: negative regulation of immune system process
GO:0045087: innate immune response
GO:0051240: positive regulation of multicellular organismal process
GO:0046649: lymphocyte activation
GO:0008284: positive regulation of cell proliferation
GO:0034728: nucleosome organization
GO:0048585: negative regulation of response to stimulus
GO:0044092: negative regulation of molecular function
GO:0008285: negative regulation of cell proliferation
GO:0048002: antigen processing and presentation of peptide antigen
GO:0070482: response to oxygen levels
GO:0031348: negative regulation of defense response
GO:0050794: regulation of cellular process
GO:0043331: response to dsRNA
GO:0032101: regulation of response to external stimulus
GO:0051239: regulation of multicellular organismal process
GO:0002757: immune response-activating signal transduction
GO:0001666: response to hypoxia

M1(−)
GO:0006952: defense response
GO:0009611: response to wounding
GO:0002684: positive regulation of immune system process
GO:0002682: regulation of immune system process
GO:0050776: regulation of immune response
GO:0048583: regulation of response to stimulus
GO:0002694: regulation of leukocyte activation
GO:0050865: regulation of cell activation
GO:0050778: positive regulation of immune response
GO:0048584: positive regulation of response to stimulus
GO:0002696: positive regulation of leukocyte activation
GO:0045087: innate immune response
GO:0050867: positive regulation of cell activation
GO:0002252: immune effector process
GO:0048518: positive regulation of biological process
GO:0002250: adaptive immune response
GO:0002443: leukocyte mediated immunity
GO:0048002: antigen processing and presentation of peptide antigen
GO:0001817: regulation of cytokine production
GO:0019884: antigen processing and presentation of exogenous antigen
GO:0006959: humoral immune response
GO:0002683: negative regulation of immune system process
GO:0002504: antigen processing and presentation of peptide or polysaccharide antigen via MHC class II
GO:0002237: response to molecule of bacterial origin
GO:0006935: chemotaxis
GO:0042330: taxis
GO:0045619: regulation of lymphocyte differentiation
GO:0007626: locomotory behavior
GO:0080134: regulation of response to stress
GO:0006956: complement activation
GO:0051707: response to other organism
GO:0042127: regulation of cell proliferation

M2a
GO:0009611: response to wounding
GO:0006935: chemotaxis
GO:0042330: taxis
GO:0008284: positive regulation of cell proliferation
GO:0007626: locomotory behavior
GO:0006952: defense response
GO:0050865: regulation of cell activation
GO:0002682: regulation of immune system process
GO:0002696: positive regulation of leukocyte activation
GO:0002694: regulation of leukocyte activation
GO:0050867: positive regulation of cell activation
GO:0048583: regulation of response to stimulus
GO:0002684: positive regulation of immune system process
GO:0042127: regulation of cell proliferation
GO:0009725: response to hormone stimulus

M2b
GO:0006952: defense response
GO:0009611: response to wounding
GO:0006935: chemotaxis
GO:0042330: taxis
GO:0051707: response to other organism
GO:0007626: locomotory behavior
GO:0009615: response to virus
GO:0002684: positive regulation of immune system process
GO:0048583: regulation of response to stimulus
GO:0002682: regulation of immune system process
GO:0042127: regulation of cell proliferation
GO:0009617: response to bacterium
GO:0002237: response to molecule of bacterial origin
GO:0007267: cell-cell signaling
GO:0050865: regulation of cell activation
GO:0008284: positive regulation of cell proliferation
GO:0050776: regulation of immune response
GO:0002694: regulation of leukocyte activation
GO:0050867: positive regulation of cell activation
GO:0048584: positive regulation of response to stimulus
GO:0032101: regulation of response to external stimulus
GO:0002696: positive regulation of leukocyte activation
GO:0048518: positive regulation of biological process
GO:0006959: humoral immune response
GO:0080134: regulation of response to stress
GO:0031497: chromatin assembly
GO:0034728: nucleosome organization
GO:0002250: adaptive immune response
GO:0002252: immune effector process
GO:0048585: negative regulation of response to stimulus
GO:0002683: negative regulation of immune system process
GO:0050778: positive regulation of immune response
GO:0045321: leukocyte activation
GO:0007165: signal transduction
GO:0001817: regulation of cytokine production
GO:0045087: innate immune response
GO:0034097: response to cytokine stimulus
GO:0010941: regulation of cell death
GO:0070482: response to oxygen levels
GO:0048522: positive regulation of cellular process
GO:0051239: regulation of multicellular organismal process
GO:0055082: cellular chemical homeostasis
GO:0031348: negative regulation of defense response
GO:0051240: positive regulation of multicellular organismal process
GO:0030595: leukocyte chemotaxis
GO:0002443: leukocyte mediated immunity
GO:0008285: negative regulation of cell proliferation
GO:0046649: lymphocyte activation
GO:0060326: cell chemotaxis
GO:0031349: positive regulation of defense response
GO:0001666: response to hypoxia

M2c
GO:0009611: response to wounding
GO:0002682: regulation of immune system process
GO:0006952: defense response
GO:0048583: regulation of response to stimulus
GO:0002684: positive regulation of immune system process
GO:0050776: regulation of immune response
GO:0006959: humoral immune response
GO:0050867: positive regulation of cell activation
GO:0002696: positive regulation of leukocyte activation
GO:0006935: chemotaxis
GO:0042330: taxis
GO:0050865: regulation of cell activation
GO:0051094: positive regulation of developmental process
GO:0048585: negative regulation of response to stimulus
GO:0002694: regulation of leukocyte activation
GO:0045087: innate immune response
GO:0045321: leukocyte activation
GO:0050778: positive regulation of immune response
GO:0007165: signal transduction
GO:0048518: positive regulation of biological process
GO:0045597: positive regulation of cell differentiation
GO:0048584: positive regulation of response to stimulus
GO:0007626: locomotory behavior
GO:0002250: adaptive immune response
GO:0002252: immune effector process
GO:0050793: regulation of developmental process
